# Some like it cold: Temperature‐dependent habitat selection by narwhals

**DOI:** 10.1002/ece3.6464

**Published:** 2020-07-22

**Authors:** Mads Peter Heide‐Jørgensen, Susanna B. Blackwell, Terrie M. Williams, Mikkel Holger S. Sinding, Mikkel Skovrind, Outi M. Tervo, Eva Garde, Rikke G. Hansen, Nynne H. Nielsen, Mạnh Cường Ngô, Susanne Ditlevsen

**Affiliations:** ^1^ Greenland Institute of Natural Resources Copenhagen Denmark; ^2^ Greeneridge Sciences Inc. Santa Barbara CA USA; ^3^ Center for Ocean Health – Long Marine Laboratory University of California‐Santa Cruz Santa Cruz CA USA; ^4^ Smurfit Institute of Genetics Trinity College Dublin Dublin Ireland; ^5^ GLOBE Institute University of Copenhagen Copenhagen Denmark; ^6^ Greenland Institute of Natural Resources Nuuk Greenland; ^7^ Data Science Laboratory Department of Mathematical Sciences University of Copenhagen Copenhagen Denmark

**Keywords:** buzzing, deep diving, East Greenland, high Arctic, oceanography, satellite tracking, thermal homeostasis

## Abstract

The narwhal (*Monodon monoceros*) is a high‐Arctic species inhabiting areas that are experiencing increases in sea temperatures, which together with reduction in sea ice are expected to modify the niches of several Arctic marine apex predators. The Scoresby Sound fjord complex in East Greenland is the summer residence for an isolated population of narwhals. The movements of 12 whales instrumented with Fastloc‐GPS transmitters were studied during summer in Scoresby Sound and at their offshore winter ground in 2017–2019. An additional four narwhals provided detailed hydrographic profiles on both summer and winter grounds. Data on diving of the whales were obtained from 20 satellite‐linked time‐depth recorders and 16 Acousonde™ recorders that also provided information on the temperature and depth of buzzes. In summer, the foraging whales targeted depths between 300 and 850 m where the preferred areas visited by the whales had temperatures ranging between 0.6 and 1.5°C (mean = 1.1°C, *SD* = 0.22). The highest probability of buzzing activity during summer was at a temperature of 0.7°C and at depths > 300 m. The whales targeted similar depths at their offshore winter ground where the temperature was slightly higher (range: 0.7–1.7°C, mean = 1.3°C, *SD* = 0.29). Both the probability of buzzing events and the spatial distribution of the whales in both seasons demonstrated a preferential selection of cold water. This was particularly pronounced in winter where cold coastal water was selected and warm Atlantic water farther offshore was avoided. It is unknown if the small temperature niche of whales while feeding is because prey is concentrated at these temperature gradients and is easier to capture at low temperatures, or because there are limitations in the thermoregulation of the whales. In any case, the small niche requirements together with their strong site fidelity emphasize the sensitivity of narwhals to changes in the thermal characteristics of their habitats.

## Graphical abstract

Narwhals avoid areas with warm temperatures. Both their prey capturing and diving behavior show their clear preference for cold habitats. They preferentially select habitats where the temperature ranges between 0 and 1°C. Photo: Carsten Egevang

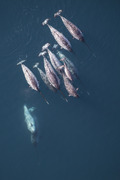

## 1 INTRODUCTION

Over the past 30 years, sea ice in the Arctic has declined by 3%–4% per decade, making the Arctic the area experiencing the most rapid degradation due to climate change (Parkinson & Cavalieri, 2002, 2008; Serreze & Stroeve, 2015; Stroeve, Schroder, Tsamados, & Feltham, 2018). At the same time, the ocean has warmed on a global scale by an average of 0.11°C per decade from 1971 to 2010 (Pachauri, Qin, & Stocker, 2013). Increasing sea temperatures and reduction in sea ice are expected to modify the niches of Arctic marine apex predators although robust niche definitions are missing for most species (Chambault et al., 2018; Laidre et al., 2008). While increasing sea temperatures may result in range expansions for species that travel between temperate and polar waters, the range of Arctic species is conversely expected to decline leading to major changes in their habitat use (Simmonds & Isaac, 2007; Tynan & DeMaster, 1997).

The narwhal is a high‐Arctic medium‐sized cetacean that makes annual migrations from coastal areas in front of glaciers to offshore deep‐water areas in Baffin Bay, the North Water, and the Greenland Sea (Heide‐Jørgensen et al., 2015; Kenyon, Yurkowski, Orr, Barber, & Ferguson, 2018; Watt, Orr, & Ferguson, 2017). The various populations or stocks of narwhals are usually delineated based on their summer grounds (Heide‐Jørgensen, Richard, Dietz, & Laidre, 2013; Watt et al., 2019). In East Greenland, narwhals in the Scoresby Sound fjord system are considered isolated from neighboring populations that summer north or south of Scoresby Sound (Heide‐Jørgensen et al., 2015).

Narwhals routinely travel through water masses that are critical for the circulation in the North Atlantic. Glacial melt at the narwhals' summer grounds in Greenland increases the inflow of freshwater which then pushes the border of warm water masses south in the North Atlantic (Hunt et al., 2015). Large‐scale freshwater anomalies have been observed in past decades and are predicted to occur with increasing frequency with global warming (Rahmstorf et al., 2015). This will maintain the cold‐water influx to narwhal habitats but at the same time there is a general warming of the sea surface throughout the North Atlantic (Alexander et al., 2018) and at several of the narwhal summer grounds (Chambault et al., in review).

Narwhals appear to avoid areas with inflow of warm Atlantic water (>4°C) in the Arctic, and this likely determines both their migrations and habitat selection (Chambault et al., in review). Modeling of narwhal habitat selection has traditionally been conducted with correlative models including data derived from satellite‐tracked whales, in which positions and diving depths are related to physical parameters (sea ice, bathymetry, etc.) or potential prey concentrations (Kenyon et al., 2018; Laidre, Heide‐Jørgensen, Jørgensen, & Treble, 2004; Watt et al., 2017). Based on this, wide areas of the Arctic are available as potential narwhal habitats, but are currently devoid of the whales. This discrepancy may be due in part to the limited understanding of the biological importance of potential foraging habitats, thus leading to erroneous assumptions about niche selection by narwhals. Also, the extrapolations of correlative habitat models to future climate scenarios are exceptionally risky without empirical data on niche selection. A more mechanistic niche description comprising environmental and biotic factors that affect the fitness of the whales is needed to understand the interactions between narwhals and their rapidly changing environment (e.g., Kearney, 2006). This is clearly a difficult task for cetaceans in general, and for narwhals in particular, as they prefer to live in logistically extremely challenging environments. Energetics‐based niche modeling requires more data on metabolic and nutritional requirements of narwhals than are currently available. One improvement is to include the effects of changes in sea temperatures in niche modeling for narwhals. This is especially important for this Arctic marine mammal as changing sea temperatures may affect both the narwhal's homeothermy and its chances for encountering and capturing prey. It has also been shown for other cetaceans that sea temperatures play an important role in niche definition including the foraging success, survival, and distribution of the whales (Chambault et al., 2018; Derville et al., 2019; Lambert et al., 2014; Owen, Jenner, Micheline‐Nicole, McCauley, & Andrews, 2018; Owen et al., 2018; Whitehead, McGill, & Worm, 2008; Wild et al., 2019).

Current models predict that sea temperatures will undergo marked changes in the Arctic both with reduced albedo from loss of sea ice and changing patterns of ocean circulation. As an endemic polar species with a limited distribution in the Atlantic part of the Arctic, the narwhal may be particularly susceptible to these changes. Furthermore, narwhals are considered highly sensitive to climate change due to lack of plasticity in movement patterns, low population size, low diet variability, and low genetic diversity (Laidre et al., 2008; Louis et al., 2020; Westbury, Petersen, Garde, Heide‐Jørgensen, & Lorenzen, 2019).

In this study, we test the narwhal's vulnerability to changing climate conditions, by examining the narwhal's dependence on a narrow temperature niche in East Greenland. Four independent data sets obtained by use of satellite telemetry and biologging methods were used to illustrate the habitat usage/selection by narwhals. Data from CTD transmitters were used to characterize the habitat's oceanography, Fastloc‐GPS transmitters showed the spatial dispersal of the whales, acoustic tags provided data on depth and temperature of buzzing activities, time‐depth recorders indicated the preferred diving depths while satellite‐linked time‐depth recorders provided concatenated data on diving activity.

## 2 MATERIALS AND METHODS

### 2.1 Study area

The Scoresby Sound fjord system (hereafter Scoresby Sound) in East Greenland is the summer residence for an isolated population of narwhals. The fjord system is about 350 km long with many side branches of smaller fjords around one large island: Milne Land. The detailed bathymetry of the fjord system is not well known but most of the inner part of the fjords have depths that range down to 1,000 m or deeper (Digby, 1953; Ryder, 1895). Extensive shallow areas with slightly sloping water depths are found in the northwestern part along Jameson Land. There are 12 active glaciers that feed into the fjord system supplemented by an inflow from the cold East Greenland current in the northern part of the entrance to the fjord system (Digby, 1953). The main current out of Scoresby Sound is in the southern part of the entrance. Sea ice forms in October in the inner parts of the fjord system, and by December, the entire fjord is ice‐covered. The sea ice persists through June; however, an open water polynya is present throughout the winter at the opening of the Scoresby Sound fjord system.

### 2.2 Study design

Four different approaches were used to illustrate the habitat usage of narwhals. Position data from 2 years of satellite tracking (August 2017–March 2019) were combined with oceanographic data to spatially identify the critical temperature ranges used by narwhals. The oceanographic data were collected independently by narwhals instrumented with CTD transmitters in the same 2 years. Archival tags recording acoustic activity by the whales in addition to depth and temperature provided information on the depth and temperature of potential foraging events in 2013–2018. Archival time‐depth recorders showed the preferred diving depths, and satellite‐linked time‐depth recorders deployed in 2010–2013 provided concatenated data on diving activity (Table 1). The summer period was defined as August through September and the winter as December through March. The migration corridors used in October–November are probably not as important as the areas where the whales are more stationary and were therefore not included in the analysis.

**TABLE 1:** List of tagged whales

Year	Sampling period	Instrument	IDNO	Sex	Length (cm)	Tusk (cm)	Sample size
2013	8–11 Aug	Acou/MM1	ACU011	F	420	–	245,586 s
2014	11–12 Aug	Acou/MM2	ACU023	F	390	–	35,964 s
2014	11–15 Aug	Acou/MM3	ACU011	F	341	–	370,006 s
2015	15–19 Aug	Acou/MM4	ACU011	F	380	–	297,602 s
2016	24–28 Aug	Acou/MM5	ACU023	F	360	–	369,090 s
2016	24–31 Aug	Acou/MM6	ACU027	M	372	74	690,241 s
2017	11–19 Aug	Acou/MM7	ACU027	M	497	200	727,440 s
2017	11–15 Aug	Acou/MM8	ACU032	M	457	220	368,700 s
2017	22–31 Aug	Acou/MM9	ACU028	F	393	–	745,050 s
2017	24–25 Aug	Acou/MM10	ACU031	M	330	40	122,580 s
2018	23 Aug–1 Sept	Acou/MM11	ACU031	M	487	207	709,273 s
2018	23–30 Aug	Acou/MM12	ACU028	M	460	>157	752,577 s
2018	23 Aug–1 Sept	Acou/MM13	ACU032	M	436	136	948,885 s
2018	24–29 Aug	Acou/MM14	ACU027	M	410	83	400,305 s
2018	24–29 Aug	Acou/MM15	ACU023	M	470	167	370,080 s
2018	25 Aug–2 Sept	Acou/MM16	ACU011	M	409	>73	368,154 s
2017	22 Aug–15 Nov	CTD	24639	F	393	–	137 casts
2017–18	23 Aug–3 Feb	CTD	37282	M	430	193	313 casts
2018–19	25 Aug–24 Jan	CTD	20696	M	402	125	293 casts
2018	26 Aug–11 Feb	CTD	21793	M	380	97	320 casts
2017	11 Aug–19 Dec	FastLoc	20165	M	454	195	4,298 pos.
2017–18	11 Aug–13 Mar	FastLoc	168435	M	497	200	5,189 pos.
2017	11 Aug–14 Nov	FastLoc	22853	M	457	220	1,842 pos.
2017	22 Aug–12 Dec	FastLoc	22849	M	477	198	751 pos.
2017–18	24 Aug–29 May	FastLoc	20162	F	379	–	4,417 pos.
2017	24 Aug–26 Aug	FastLoc	168434	M	330	40	284 pos.
2018–19	23 Aug–23 Jan	FastLoc	168437	M	487	207	6,117 pos.
2018	23 Aug–3 Sep	FastLoc	21791	M	460	157 (broken)	1,874 pos.
2018–19	23 Aug–2 Jan	FastLoc	20158	M	436	136	6,018 pos.
2018–19	24 Aug–6 Mar	FastLoc	20160	M	410	83	5,586 pos.
2018–19	24 Aug–13 Feb	FastLoc	168433	M	470	167	6,235 pos.
2018–19	25 Aug–9 Jan	FastLoc	168436	M	409	73 (broken)	6,215 pos.
2010	2 Sep–29 Dec	SLTDR	3960	M	400	90	1,07 6‐hr hist.
2010–11	22 Aug–17 Feb	SLTDR	3963	F	395		129 6‐hr hist.
2010–11	2 Sep–15 Feb	SLTDR	3964	M	385	104	109 6‐hr hist.
2010–11	4 Sep–12 Jul	SLTDR	6335	F	395		170 6‐hr hist.
2010	4 Sep–13 Nov	SLTDR	93093	M	275	9	46 6‐hr hist.
2010–11	4 Sep–9 Jun	SLTDR	93094	F	415		249 6‐hr hist.
2011–12	12 Aug–6 Mar	SLTDR	6336	F	315		22 6‐hr hist.
2011–12	12 Aug–9 Jan	SLTDR	7926	M	407	100	8 6‐hr hist.
2011–12	12 Aug–18 Jan	SLTDR	20162	M	392	98	15 6‐hr hist.
2011–12	13 Aug–3 Mar	SLTDR	93098	M	290	23	15 6‐hr hist.
2011	16 Aug–22 Oct	SLTDR	93095	M	370	83	7 6‐hr hist.
2011–12	19 Aug–12 May	SLTDR	93101	M	453	170	10 6‐hr hist.
2011–12	19 Aug–18 Mar	SLTDR	10946	M	364	60	9 6‐hr hist.
2012	19 Aug–3 Dec	SLTDR	21792	F?	280	–	327 6‐hr hist.
2012–13	23 Aug–10 Apr	SLTDR	21791	M	440	150	831 6‐hr hist.
2013	8 Aug–2 Sep	SLTDR	93096	F	420	–	20 6‐hr hist.
2013	13 Aug	SLTDR	3965	M	400	106	7,182,819 s
2013–14	17 Aug–31 May	SLTDR	93097	F	385	–	141 6‐hr hist
2013	20 Aug–24 Nov	SLTDR	20685	M	327	23	167 6‐hr hist
2013	17–31 Aug	SLTDR	93102	M	417	109	48 6‐hr hist
2014	11 Aug–28 Dec	SLTDR	3962	M	414	100	495 6‐hr hist
2016	24 Aug–17 Oct	SMRU CTD	27262	M	325	53	187 casts

Note

Acousonde (Acou) tags provided data on acoustics, depth, and temperature; CTD tags provided data on position and oceanographic profiles (temperature and salinity at depth); FastLoc tags provided high‐resolution (GPS‐based) positions; SLTDR tag provided coarse data on diving activity in bins sampled in 6‐hr periods, except for IDNO 3965 that provided a continuous record of dives at a resolution of 1 m for every second; and the SMRU CTD tag provided data on target depths of dives. Sample size is given in seconds (s) for Acousonde tags, in casts for CTD tags, in number of positions (pos.) for Fastloc‐GPS transmitters, in 6‐hr histograms (6‐hr hist.) for SLTDR tags and in seconds for one SLTDR (#3965). *F* = female, *M* = male.

### 2.3 Positions of whales

Wildlife Computers (Redmond, Seattle, WA, USA) Fastloc‐GPS‐receivers and Argos transmitters (SPLASH10‐BF‐2380) were mounted on the back of twelve whales across 2017 and 2018 (see Table 1) with three 8‐mm delrin nylon pins secured with nylon washers and bolts on each end, following instrumentation techniques used in similar studies in Canada and West Greenland (Dietz et al., 2008; Heide‐Jørgensen et al., 2003). The transmitters were programmed to collect an unrestricted number of FastLoc snapshots through August and September. In 2017, the transmitters were restricted to provide data only every seventh day in September. The Fastloc snapshots were transmitted to and relayed through the Argos Location and Data Collection System (www.argos‐system.cls.fr). Postprocessing of GPS positions was conducted through the Wildlife Computers web portal. Fastloc‐GPS is a GPS positioning system with the ability of faster acquisition of animal positions than traditional GPS (Bryant, 2007; Tomkiewicz, Fuller, Kie, & Bates, 2010). A total of 34,825 Fastloc‐GPS positions were obtained from the 12 whales (daily range between 10 and 278 positions) during August and September. The first 24 hr of positions after the release of the whales were discarded to remove possible effects of capture and tagging. To avoid the effect of different sampling intensity for each whale, a subset consisting of a random sample of ten positions per whale per day provided 290 whale‐days with positions (2,900 positions in total). These were subsampled in proportion to the number of days with positions from each whale to provide a subset of 1,000 positions and used as an unbiased estimator of the localities selected by the whales during summer. During winter (December through March), Fastloc‐GPS positions (*n* = 2,276) were acquired every seventh day from five whales and, since the whales are considered more stationary at their wintering area (Heide‐Jørgensen et al., 2015), it was decided to use one daily position for each whale (89 positions).

### 2.4 Oceanographic data

In both 2017 and 2018, two narwhals were instrumented with satellite transmitters that in addition to depth also recorded and transmitted data on in situ water temperature and salinity (Wildlife Computers Scout‐CTD‐370D, 12 × 6 × 3.5 cm, 316 g). The CTD (conductivity–temperature–depth) tags were electrode‐based, were powered by four AA lithium batteries, and had a temperature range of −3 to 40°C and a salinity range of 0–50 PSU. The resolution was 0.001 for salinity and temperature, and 0.5 m for depth. The minimum requirement for a dive was 50 m and the minimum interval between dives was set to 15 min. For each qualifying dive, the deepest point was detected, after which sampling of the various parameters was continued at 1 Hz until reaching the surface. The first dive set the baseline or the minimum depth for a dive to be recorded and the next dive had to be ten percent deeper than the baseline dive to overwrite the data. This continued until the end of the summary period, when the CTD data and the closest position data were processed into Argos messages for transmission. When the tag was at the surface, the measured environmental data were transmitted through Argos Data Collection and Location System. The tags were programmed to capture the deepest profile in every 12‐hr period, that is, a total of two profiles per day, and were set to transmit the profile repeatedly 12 times to increase the chance that a given profile would be received by an Argos satellite during the following 12‐hr period. The tags were mounted in a similar way as the Fastloc‐GPS transmitters mentioned above. Data from the CTD casts were collected at standard depths following Levitus World Ocean Atlas 1994 (WOA94, 0, 10, 20, 30, 50, 75, 100, 125, 150, 200, 250, 300, 400, 500, 600, 700, 800, 900, 1,000 m, etc.) standard depths and each cast had an associated Fastloc‐GPS snapshot, providing only two positions per day from these tags. The data from these tags were used for characterizing the sea temperatures and salinities in Scoresby Sound and adjacent offshore areas, and the four whales that carried the CTD tags were not used for the analyses of the selection of thermal habitats.

The temperature data from the four CTD tags were averaged across the 2 years for each grid cell. For the summer data, the number of whale positions and the average ocean temperatures were calculated in a grid net of approximately 10 × 10 km. Fewer positions from the whales were obtained during winter, and a coarser grid net of 25 × 25 km for averaging the CTD casts was chosen for this season.

### 2.5 Acoustic recordings

Sixteen narwhals were instrumented with Acousonde™ acoustic tags (Table 1, www.acousonde.com), whose float had been modified to accommodate an Argos transmitter (Wildlife Computers SPOT5) in addition to a VHF transmitter (ATS Telemetry). The Acousonde recorders were attached to the skin with suction cups, on the rear half of the animal, to the side of the dorsal ridge, and released from the whales after four to ten days (see Blackwell et al., 2018). They were subsequently located and picked up at sea with Argos and VHF transmitters. A custom‐written buzz detector (MATLAB, The MathWorks, Inc.) was used to identify buzzes in the records and all potential buzzes were verified manually in 13 of the Acousonde recordings with sufficient quality of the acoustic data. As was done for the GPS positions, the first 24 hr of acoustic data were excluded. In addition to acoustic sampling the Acousonde also provided data on depth (precision 0.5 m) and temperature (precision of 0.01°C) every one second. Since the temperature sensor on the Acousonde is embedded in epoxy, there is a delay in the temperature readings relative to the depth. It is therefore necessary to correct the measured temperatures to obtain the actual temperature values and a detailed description of how the corrected temperatures were obtained is provided in Appendix 1. The predicted occurrence of buzzes in relation to depth and temperature was modeled with generalized linear mixed‐effects models with a Poisson response distribution with log‐link (function *glmer*, package “lme4” (Bates, Maechler, Bolker, & Walker, 2015) in R 3.5.). The best model based on the lowest AIC score included temperature (nonlinearly, as second‐order polynomial variable), depth (nonlinearly with natural splines with three degrees of freedom), and sex as fixed effects. Individual whales were included as a random effect. In order to distinguish between different layers of water column stratification and to isolate the layer known to have the highest buzzing activity (Blackwell et al., 2018), three depth strata were used for the modeling (0–100 m, 100–300 m, >300 m). This allowed the model to predict the effect of temperature on the buzzing rate separately for each depth stratum. The effect of sex on the temperature preference for buzzing was tested by comparing AIC and predicted values for models with and without sex.

### 2.6 Diving activity

Dive information from several different types of instruments was obtained from a total of 38 whales. The Acousonde tags provided whole dive profiles sampled at 1 Hz. The time spent at depth categories that match the Levitus depth bins was calculated for the 16 Acousonde tags deployed on narwhals for 4–8 days (see Blackwell et al., 2018) and for one narwhal tagged in 2013 with a satellite‐linked time‐depth recorder (SLTDR) that provided an exceptionally long record of 83 days at a 1 s resolution for depth readings (see Heide‐Jørgensen et al., 2015; Ngô, Heide‐Jørgensen, & Ditlevsen, 2019). One whale instrumented with a CTD tag manufactured by Sea Mammal Research Unit in 2016 provided data (65 days) on the target depths of the diving activity of the whale.

Another 20 narwhals instrumented with SLTDRs and tracked between 2010 and 2014 (see Watt, Orr, Heide‐Jørgensen, Nielsen, & Ferguson, 2015 and Table 1) provided data on the mean depth of the deepest dives during 6‐hr periods. During summer nine of the whales with SLTDRs also provided data on the time spent at coarse depth intervals (0–200 m; 200–500 m; 500–800 m; 800–1100 m) but only five of the whales provided data during the winter period.

## 3 RESULTS

### 3.1 Summer

**FIGURE 1:**
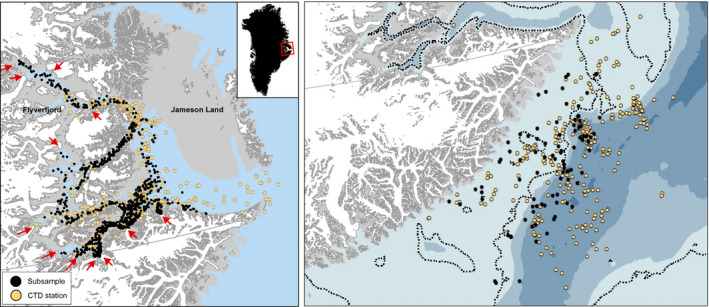
Left: The Scoresby Sound fjord complex with active glaciers (red arrows) and positions from 12 narwhals in Scoresby Sound (black dots). The positions were downsampled to 10 positions per day, which again was resampled to 1,000 positions. Positions of CTD profiles collected by four whales in August–September 2017 and 2018 are shown below the black dots as yellow dots (drawn below the black dots). An inset shows the study area in East Greenland. Right: Winter (December through March) positions of whales (black dots) and positions of CTD casts (yellow dots). Dotted line shows the 300 m depth contour

The whales used the entire Scoresby Sound fjord complex in the original data set of positions, and in the resampled data set, only one side fjord (Flyverfjord) was not included (Figure 1). The distribution of the resampled positions during summer showed that only 110 out of the 1,000 positions were located within 5 km from the nearest active glacier.

**FIGURE 2:**
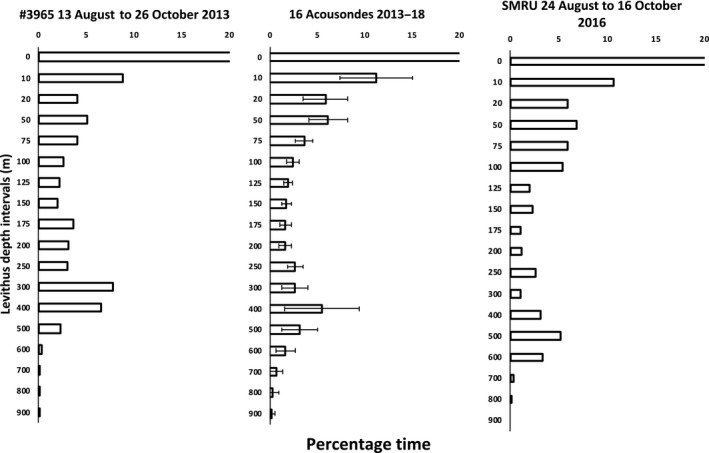
Cumulative time spent at depth in Scoresby Sound, presented as percentages for one narwhal instrumented with a SLTDR (left) with an exceptionally long record (#3963, 83 ds) and the mean of 16 narwhals instrumented with Acousondes (middle, 4–8 ds). Time spent at 0–10 m (not shown) was 44% for #3963 and 47% (*SD* = 9.62) for the Acousonde data. One *SD* is shown for the mean of the Acousonde data. Note the increased attendance to depths between 300 and 500 m. The right figure shows the percentages of the target depths (maximum depth reached during 915 dives) for one whale instrumented with a SMRU transmitter. For this, whale about 43% of the dives targeted depths < 10 m

The cumulative time spent at depth is the best predictor of the most important depths for the whales as it includes the time dedicated to foraging as well as time spent transiting to the target depths. The high‐resolution data on diving activity from the archival tags (16 Acousondes and 1 retrieved SLTDR) showed that the whales spent more time in depth bins between 250 and 600 m compared to both shallower (100–250 m) and deeper (>600 m) depth bins (Figure 2). The time spent between 10 m and 250 m's depth primarily consisted of transit time between the surface and depths below 250 m; however, the SMRU‐tag, that counted the number of dives to specific target depths, showed a bimodal distribution with most dives targeting depths < 100 m and between 250 and 600 m. The SLTDRs deployed in 2010–2013 showed that the mean of the deepest dive depths for all 6‐hr periods was 291 m (*SD* = 55) during summer. It was obvious from all tags that few dives targeted depths > 600 m during summer and that a conspicuous number of dives targeted depths between 300 and 600 m which is also the preferred depths for buzzing activity (Blackwell et al., 2018).

**FIGURE 3:**
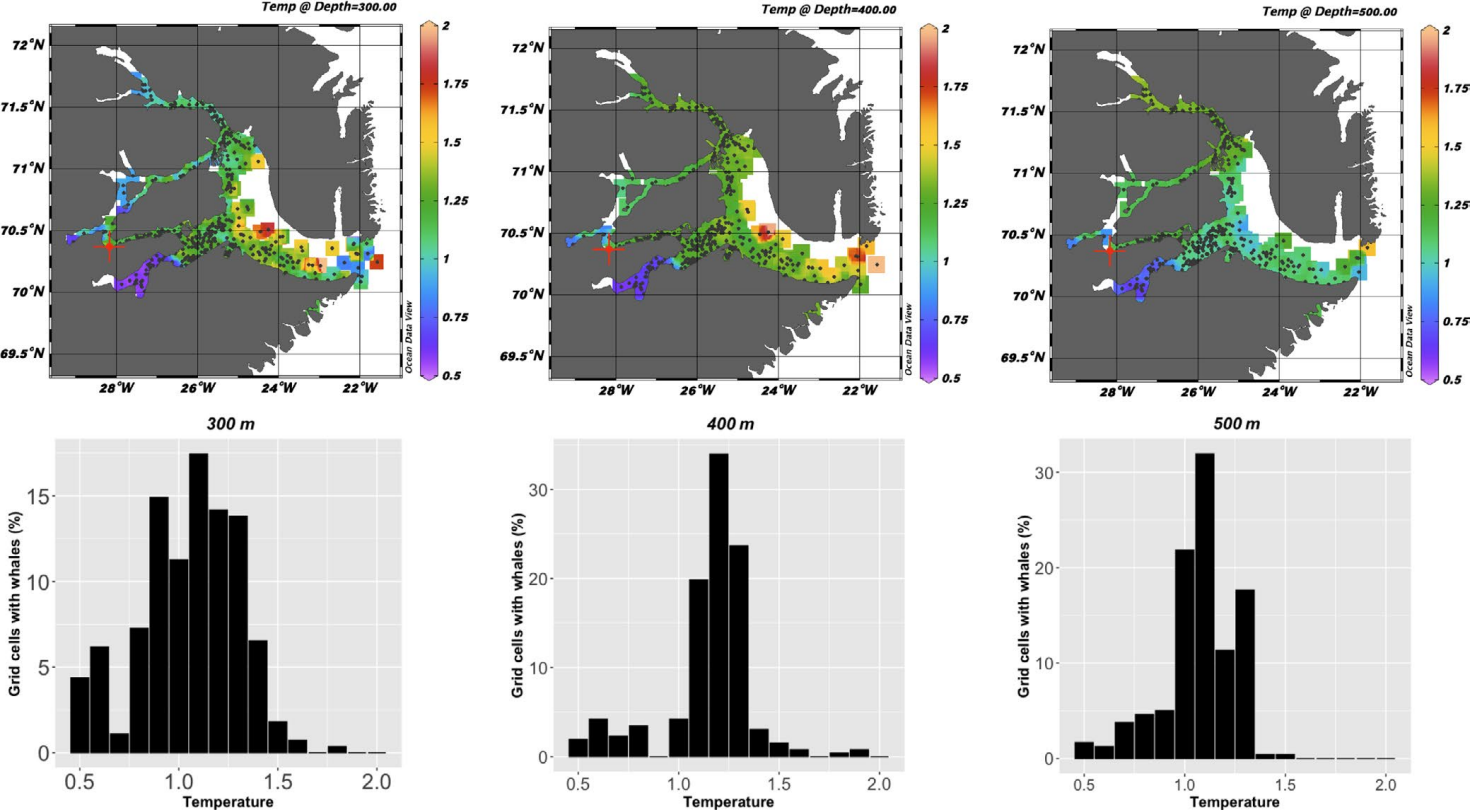
Temperature at depth at the locations (grid cells) selected by the whales during summer (August–September). The upper panel shows the isosurface distribution of temperatures at 300 m, 400 m, and 500 m depth which are the depth categories that are most targeted by the whales. Black dots show the positions of the CTD casts. The lower panel shows the percentage grid cells that were selected by the whales with the associated mean temperature in the grid cells

A total of 1,063 CTD casts were obtained from the 4 narwhals instrumented with the CTD tags of which 284 of the casts were from August to September when the whales were always inside Scoresby Sound. Thirty percent of the casts reached a maximum depth of 700 m and 5% covered the water column down to 1,000 m or deeper. The CTD profiles provided an almost complete description of the oceanographic conditions in the Scoresby Sound fjord complex in 2017 and 2018 (Figure 3).

**FIGURE 4:**
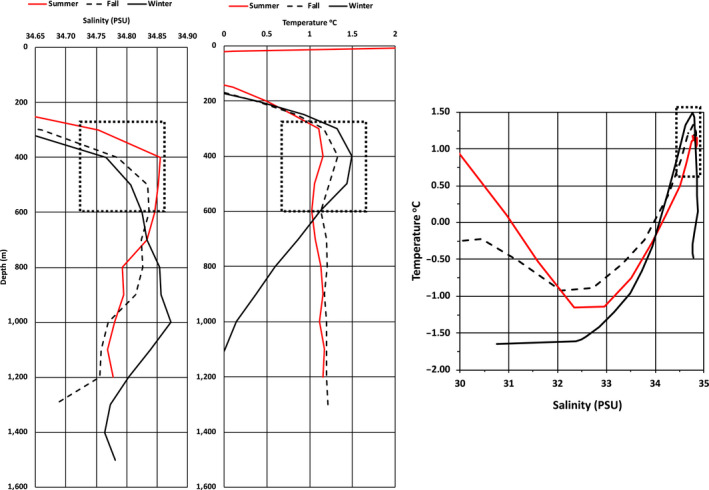
Average salinity and temperature profiles measured by the narwhals during summer, fall, and winter 2017–2018 (left) and the temperature–salinity (PSU = practical salinity unit) diagram for the three seasons (right). The square boxes indicate the T‐S regimes that were targeted by the whale's diving and buzzing activity

**FIGURE 5:**
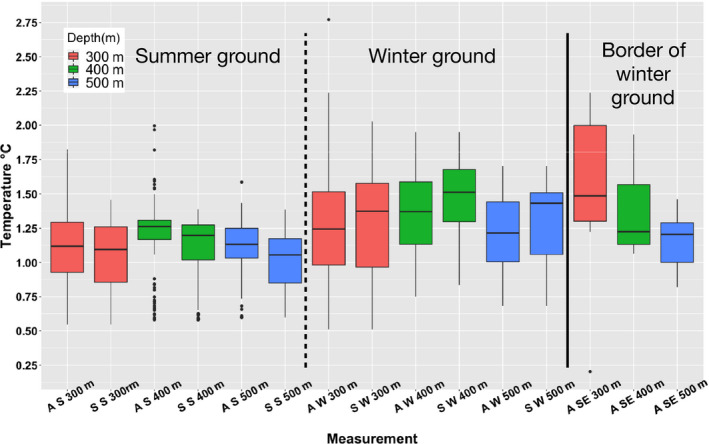
Box plots of available and selected temperatures at three depths (300–400 m, 400–500 m, 500–600 m) at the summer and winter grounds. A S = available in summer, S S = selected in summer, A W = available in winter, S W = selected in winter, A SE = available in the southeastern corner of the winter ground. None of the independent samples of whales with FastLoc transmitters selected the southeastern corner of the winter ground

The temperature at the locations that the 12 whales with Fastloc‐GPS transmitters selected for their deep diving activity (300–600 m) during summer varied between 0.6 and 1.5°C (mean 1.1°C, *SD* = 0.22), which is in the lower range of the available temperatures (0.6–2.0°C, mean = 1.2°C, *SD* = 0.23) at that depth throughout the study area as determined by the four whales with CTD transmitters that collected independent temperature and salinity profiles of the fjord system (Figures 3, 4, 5).

**FIGURE 6:**
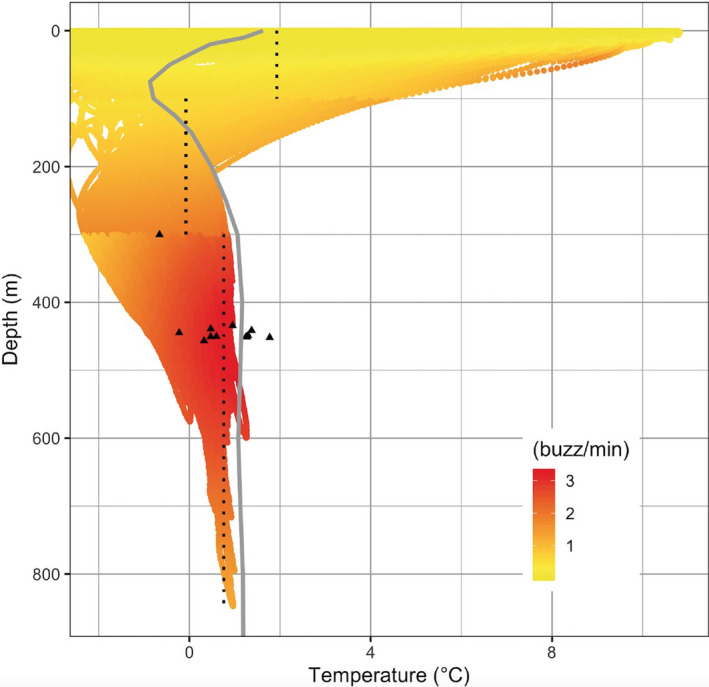
Predicted buzz rates (buzz/min) (22,519 buzzes) in relation to depth and temperature from generalized mixed‐effects model. The average temperature profile from the CTD casts is shown as the gray line. The black dotted lines mark the population estimates for the temperature with the highest predicted buzzing rates in each of the three depth categories. The estimates were 0–100 m: 1.9°C, 100–300 m −0.1°C, and >300 m: 0.7°C. The black triangles mark the individual specific estimates for the highest predicted buzzing rates (see Table 2 for details). Seven out of 12 whales selected temperatures that were lower than the average temperature. Extreme values below −2°C were omitted (<2% of the values). The number of buzzes for the three intervals is 0–100 m: 1.5 buzzes/1000 s, 100–300 m: 24.5 buzzes/1000 s, and >300 m: 47 buzzes/1,000 s. Extreme values below −2°C were omitted (<2% of the values)

Buzzing, and therefore presumably foraging activity by the whales (e.g., Miller, Johnson, & Tyack, 2004), was highest at depths below 300 m with the highest predicted buzzing rate at 0.7°C in this depth category (Figure 6, Table 2). This is slightly lower than the average temperatures available at 300–400 m (0.5–2°C, mean = 1.13°C, *SD* = 0.25) from the CTD profiles and is also supported by 7 out of 12 whales that preferred temperatures lower than the average temperature. Less than 2% of the buzzes took place at temperatures above 2°C and less than 8.5% of the buzzes were above the thermocline at ~100 m depth. Including sex or not in the model did not change the estimates of the temperature preference for buzzing activity; however, the male buzzing rate was significantly lower than the females.

**TABLE 2:** List of whales instrumented with Acousonde recorders (see Table 1 for details on individuals) with the preferred depth, temperature, and predicted maximum buzzing per rate for each of three depth categories

Whale	Depth category (m)	Depth (m)	Temperature	Predicted max. buzz rate/min
Acou/MM1	0−100	85	−3.47	0.58
Acou/MM3	0−100	51	8.39	2.75
Acou/MM4	0−100	65	7.64	3.64
Acou/MM5	0−100	98	−4.08	0.98
Acou/MM6	0−100	100	−1.94	1.27
Acou/MM7	0−100	99	−1.83	6.20
Acou/MM8	0−100	60	7.49	4.57
Acou/MM11	0−100	59	7.25	0.86
Acou/MM12	0−100	51	5.88	2.39
Acou/MM13	0−100	99	−5.34	1.65
Acou/MM14	0−100	76	−2.65	0.41
Acou/MM15	0−100	54	6.15	0.48
Acou/MM16	0−100	100	−3.91	0.06
Acou/MM1	100−300	299	−1.35	1.13
Acou/MM3	100−300	299	0.82	3.07
Acou/MM4	100−300	299	0.98	3.89
Acou/MM5	100−300	299	−3.57	1.86
Acou/MM6	100−300	299	−0.98	2.66
Acou/MM7	100−300	276	−0.87	7.44
Acou/MM8	100−300	101	5.28	3.88
Acou/MM11	100−300	299	1.41	1.53
Acou/MM12	100−300	299	1.57	2.70
Acou/MM13	100−300	102	9.53	1.72
Acou/MM14	100−300	299	−0.29	0.84
Acou/MM15	100−300	299	1.26	0.84
Acou/MM16	100−300	106	3.48	0.06
Acou/MM1	300−850	457	0.32	1.55
Acou/MM3	300−850	434	0.95	4.65
Acou/MM4	300−850	451	1.25	5.87
Acou/MM5	300−850	451	0.60	1.89
Acou/MM6	300−850	445	−0.23	3.20
Acou/MM7	300−850	300	−0.66	6.91
Acou/MM8	300−850	441	1.37	4.32
Acou/MM11	300−850	451	1.27	2.14
Acou/MM12	300−850	452	1.77	3.83
Acou/MM13	300−850	451	0.47	1.70
Acou/MM14	300−850	439	0.47	1.18
Acou/MM15	300−850	449	1.30	1.21

Note

The unrealistic temperatures below −2°C, that occur in the upper two depth ranges, are due to the modeled corrections of the Acousonde temperature measurements.

### 3.2 Winter

**FIGURE 7:**
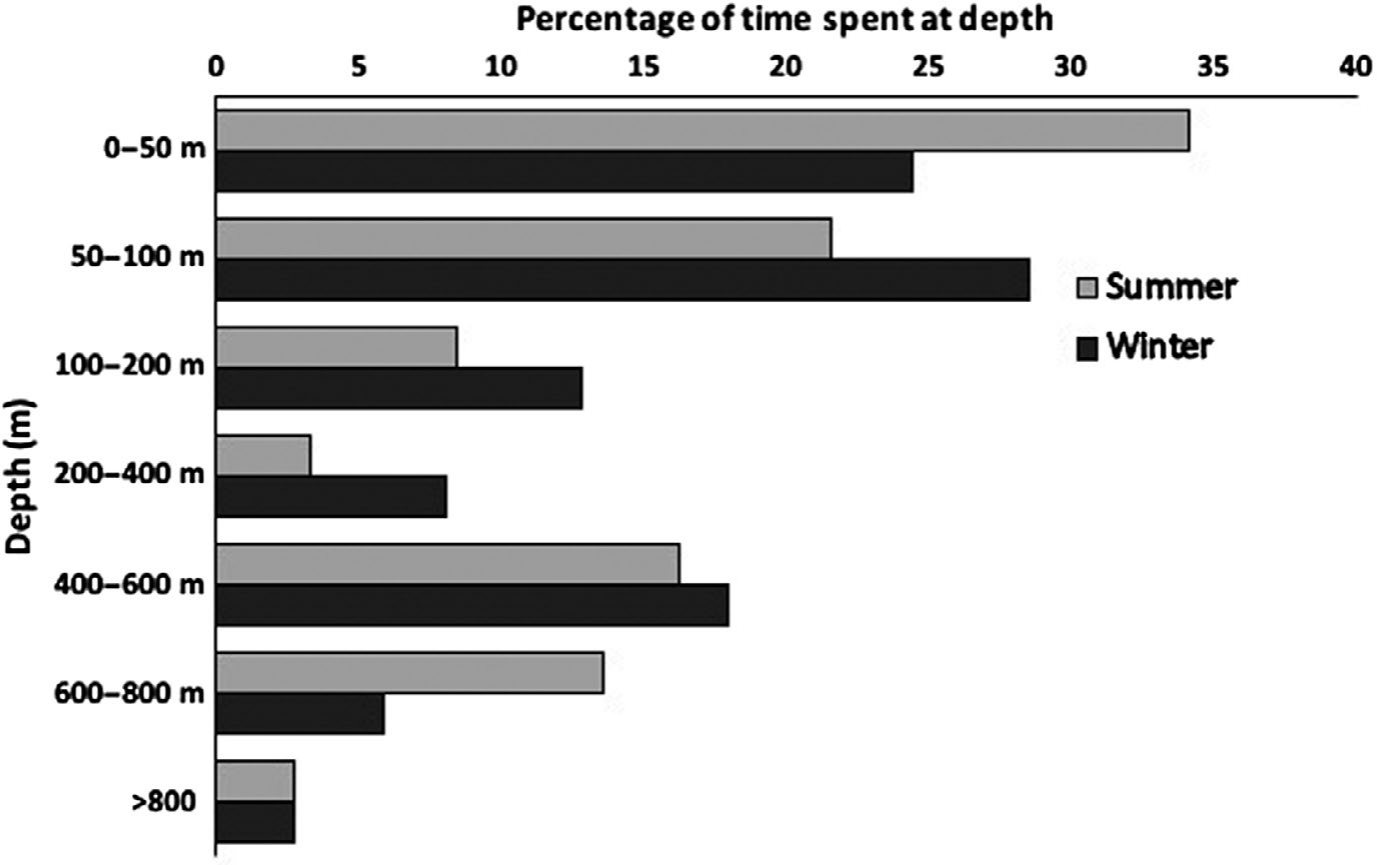
Time spent in different depth categories for two narwhals during summer (August and September 2014) and during winter (December to March 2014–2015). Data are from SLTDRs

The resolution of the target depths for the winter diving activity was less precise than for the summer diving activity, due to the use of long‐term tags (SLTDR tags) that provided binned data on diving activity rather than archived data with all depth readings (Acousonde tags). The SLTDRs deployed in 2010–2013 showed that the mean of the deepest diving depths over 6‐hr periods was 324 m (*SD* = 75) during winter for 17 narwhals tracked between 2010 and 2013. The accuracy of the binned dive data from the SLTDRs can be compared to the more detailed short‐term archival data collected during summer. The mean time spent between 200 and 600 m depth during summer was 14% (*SD* = 5.6) for the 16 Acousonde recordings and the value for similar measurements for a subset of two whales with SLTDRs for the same depth range from 2013 to 2014 was 20% (*SD* = 0.5). The difference between the binned dive data from the SLTDRs and the more detailed short‐term archival data is acceptable considering that different animals were sampled, with different instruments in different years. During winter the two whales with SLTDRs spent 26% (*SD* = 4) of their time between 200 and 600 m (Figure 7), they had more diving activity below 600 m during summer and there was generally little diving activity (<4%) targeting depths >800 m during both winter and summer.

**FIGURE 8:**
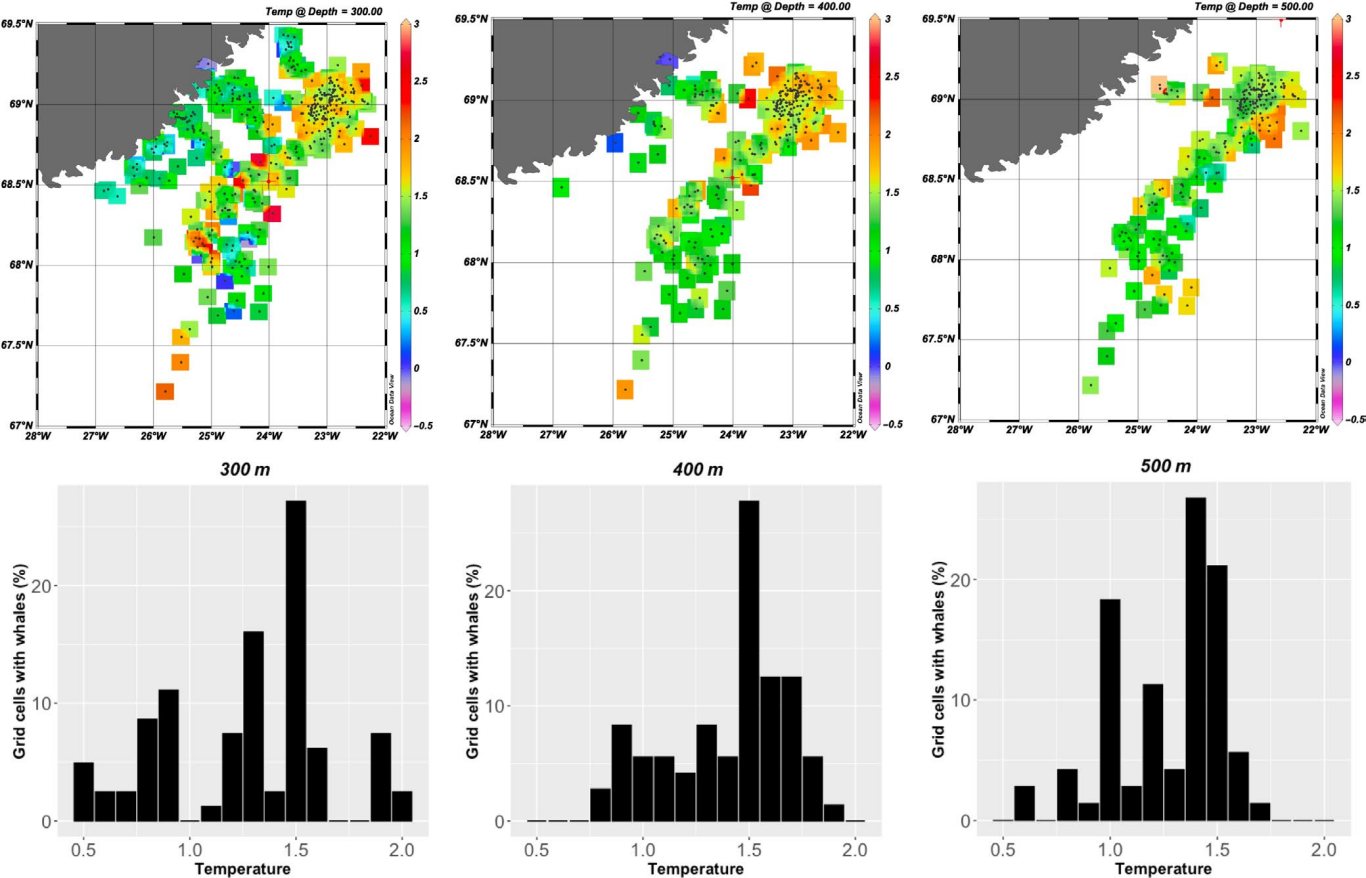
The temperature at depth at the locations (grid cells) selected by the whales during winter (December–March). The upper panel shows the isosurface distribution of temperatures at 300 m, 400 m, and 500 m depth which are the depth categories that are most commonly targeted by the most diving activity by the whales. Black dots show the positions of the CTD casts. The lower panel shows the percentage grid cells that were selected by the whales with the associated mean temperature in the grid cells

The 263 oceanographic profiles obtained from the offshore wintering area covered the main winter distribution of the whales (Figure 8). The vertical coverage included 38% of the casts reaching 700 m and 25% that went to 1,000 m or deeper. The mean temperature for the depths selected by the narwhals was similar for the 300–400 m (1.3°C, *SD* = 0.43, Figure 5) and 500–800 m depth ranges (1.0°C, *SD* = 0.56). Profiles from the easternmost CTD casts showed slightly higher temperatures than those selected by the whales.

The overall selection of the water column properties at the target depths of narwhal diving in both summer and winter shows a narrow range of both temperature (0.5–1.5°C) and salinity (34.72–34.86PSU) values compared to the available habitats (Figure 4). In winter, there appeared to be a secondary thermocline below 400 m but no obvious change in salinity.

## 4 DISCUSSION

Although Scoresby Sound is the world's largest fjord system (~14,000 km^2^), very little information is available on the oceanographic conditions in the area and few in situ CTD profiles have been collected and all are outdated (Digby, 1953; Koch, 1945; Ryder, 1895; Ussing, 1934). Even though the sampling was coarse both spatially (grid cells) and vertically (depth intervals), the four narwhals equipped with CTD sensors provided the most complete hydrographic coverage of not just Scoresby Sound but of any narwhal summering locality. The profiles were restricted vertically by the maximum depths of the dives of the whales but with several profiles to 1,000 m most of the water column was sampled. Only areas in the eastern part of the fjord along Jameson Land were not sampled by the whales and were clearly not part of the whales' habitat as also seen in an earlier tracking study (Heide‐Jørgensen et al., 2015). A relatively low proportion (<15%) of the whale positions were obtained from areas close (<5 km) to glacier fronts. This indicates that the narwhal's affinity for glacier fronts documented for other areas (Laidre et al., 2016) does not seem to apply for Scoresby Sound. One reason could be the prevalence of cold water throughout the fjord system rather than just in front of glaciers as seen in other coastal areas.

The whales exited Scoresby Sound during October–November well ahead of the formation of fast ice (cf. Heide‐Jørgensen et al., 2015). From December through March, the whales were concentrated on their wintering ground over the continental shelf. The spatial coverage of the oceanographic sampling in the offshore area reflected the more stationary behavior of the whales during winter. The wintering ground was centered between 68°N and 70°N, to the west by the coastline, and to the east by the edge of the continental shelf. Further east, 80–120 km from the coast in the Greenland Sea, the whales would be in an unsuitable habitat with warmer Atlantic water from the Irminger Current (Rudels, Fahrbach, Meincke, Budéus, & Eriksson, 2002; Våge, Papritz, Håvik, Spall, & Moore, 2015; Våge et al., 2013). Even though the whales preferred dense, high salinity waters, they clearly stayed away from areas with water temperatures > 2°C while at the same time avoided temperatures < 0.5°C.

During presumed foraging, there was a clear selection of water depths between 300 and 600 m for the narwhals both inside Scoresby Sound and on the offshore wintering ground (see also Manh et al. 2019), which likely reflects the depth of prey layers the whales are targeting. Narwhals feed on a few species, such as polar cod (*Boreogadus saida*), Greenland halibut (*Reinhardtius hippoglossoides*), and squids (*Gonatus* sp.) that are all potentially available in Arctic waters deeper than 300 m (see Laidre & Heide‐Jørgensen, 2005). However, information on distribution and abundance of any of the key prey species is missing for Scoresby Sound and adjacent offshore areas let alone for most of the Arctic. Given the relative slow swimming speed and short duration (<20 min) of their deep dives compared to other deep diving whales (e.g., beaked whales, *Ziphius cavirostris*, Schorr, Falcone, Moretti, & Andrews, 2014, Tyack, Johnson, Soto, Sturlese, & Madsen, 2006), narwhals must be efficiently targeting abundant prey layers. It is estimated from measures with stomach temperature pills that narwhals achieve about ten prey capture events per day during summer (Heide‐Jørgensen, Nielsen, Hansen, & Blackwell, 2014). Considering the small prey items identified in stomach contents in summer, this is probably an insufficient food intake to balance the energetic needs of narwhals and it must be assumed that the prey capture rate and the biomass of prey items increase at other times of the year.

There were few oceanographic profiles outside the continental shelf, and it is well known that water from the warm Atlantic current, that is running north between Iceland and Greenland, dominates east of the shelf area, where narwhals are not known to occur (Brakstad, Våge, Håvik, & Moore, 2019; Dietz, Heide‐Jørgensen, Glahder, & Born, 1994; Jochumsen et al., 2017). Although the warmest temperatures both on the summering ground inside Scoresby Sound and on the offshore wintering ground were detected at 400–500 m depth, followed by 300–400 m and 500–600 m depths, the temperatures were still consistently below 2°C. This was clearly also the upper limit of the temperature range for the deep‐water (>300 m) buzzing activity. The main diving preference of the whales targeted areas both vertically and horizontally that met these temperature requirements.

During summer, the whales preferred areas where the temperature at the target depths was slightly colder than the water masses that were generally available in Scoresby Sound. The same was the case for the buzzing activity but no statistically significant difference in the selected and available temperatures could be detected. It may, however, be argued that the homogenous conditions of the hydrography in Scoresby Sound makes it difficult to select temperature ranges that varied much from the available temperatures. In winter, the situation was different as even though the whales selected temperatures slightly above the available temperature, the cold coastal water of polar origin was selected disproportionally relative to the large areas of warm Atlantic water further offshore.

The spatial distribution of the whales under extrinsic conditions provides a two‐dimensional description of their habitat selection (Kenyon et al., 2018; Laidre, Heide‐Jørgensen, Logsdon, et al., 2004). The buzz activity in relation to depth and temperature provides a mechanistic approach to a vertical niche definition. The buzz activity in summer and diving activity in both summer and winter was concentrated at depths where the temperatures were below 2°C. The winter diving activity was more focused on dives to slightly deeper depths than in summer with a focus on depths between 300 and 600 m. The depth range selected by the whales targeted parts of the water column with temperatures below 2°C and confirms the niche selection of the summering area.

Temperature‐dependent targeting of dive and buzz activity seems critical for narwhals when locating prey concentrations at depths, but the drivers behind this selection remain uncertain. Narwhals feed on squid and fish and some of the target prey may be more abundant at certain depths and thermal gradients, or at the observed pycnocline around ~400 m, but the prey could also be easier to catch at lower temperatures for a relatively slow‐moving whale (Williams, Noren, & Glenn, 2010). The low temperatures may also serve to ensure thermal homeostasis. Narwhals appear to have a limited ability of transferring excess heat to the environment, in part due to the lack of a prominent dorsal fin to serve as a thermal window (Doidge, 1990). This has also been suggested for another whale endemic to the Arctic, the bowhead whale, *Balaena mysticetus* (Chambault et al., 2018). The significance of these findings for thermal balance in narwhals can only be speculated upon as studies of thermoregulation in cetaceans are still in their infancy compared to pinnipeds (Nieaber, Thomton, Horning, Polasek, & Mellish, 2010; Noren, Williams, Berry, & Butler, 1999). In view of the strict thermal preference of narwhals (Figures 4 and 5), such studies are especially needed for the Arctic cetaceans that will be exposed to the most rapid increases in ocean temperatures (Alexander et al., 2018).

## 5 CONCLUSIONS

Studies of the underwater habitat selection by deep diving marine mammals are logistically and conceptually challenging. In this study, we attempted, for the first time, to integrate oceanographic profiling with diving and foraging behavior of a high‐Arctic cetacean, the narwhal, to estimate its habitat selection. Some cetaceans make seasonal migrations that cover very different habitats, for example, breeding at low latitudes and summering at high latitudes. Narwhals also migrate between summer and wintering grounds but they essentially remain within the same habitat and avoid excursions into water masses with temperatures higher than 2°C at the depths where feeding presumably occurs. The narrow temperature habitat together with the strong site fidelity confirms the sensitivity of narwhals (Laidre et al., 2008) and demonstrates their limited ability to adapt to environmental changes. A mechanistic prediction of sea temperature changes in the high Arctic suggests that several of the present narwhal habitats are changing and that the optimal habitats for narwhal will be located further north by the end of this century (Alexander et al., 2018, Chambault et al., in review; Louis et al., 2020).

## CONFLICT OF INTEREST

There are no competing interests.

## AUTHOR CONTRIBUTIONS

**Mads Peter Heide‐Jørgensen:** Conceptualization (lead); data curation (lead); formal analysis (lead); funding acquisition (lead); investigation (lead); methodology (equal); project administration (lead); resources (equal); software (equal); supervision (equal); validation (equal); visualization (equal); writing–original draft (equal); writing–review and editing (equal). **Susanna B. Blackwell:** Data curation (equal); formal analysis (equal); investigation (equal); methodology (equal); software (equal); writing–original draft (equal). **Terrie M. Williams:** Conceptualization (equal); investigation (equal); writing–original draft (equal). **Mikkel Holger S. Sinding:** Investigation (equal); resources (equal); writing–original draft (equal). **Mikkel Skovrind:** Investigation (equal); writing‐original draft (equal). **Outi M. Tervo:** Conceptualization (equal); data curation (equal); formal analysis (equal); investigation (equal); methodology (equal); software (equal); writing–original draft (equal). **Eva Garde:** Investigation (equal); writing–original draft (equal). **Rikke G. Hansen:** Investigation (equal); methodology (equal); software (equal); visualization (equal); writing–original draft (equal). **Nynne H. Nielsen:** Data curation (equal); formal analysis (equal). **Mạnh Cường Ngô:** Formal analysis (equal). **Susanne Ditlevsen:** Methodology (equal); software (equal); validation (equal); writing–original draft (equal).

## Data Availability

Data available from the Dryad Digital Repository://orcid.org/0000‐0003‐4846‐7622.

## APPENDIX 1

### Correction of temperature measurements from Acousonde recorders

A laboratory experiment was carried out with the seven Acousondes used on narwhals with concurrent actual temperature measurements by a CTD. The CTD and the Acousondes were first submersed in water at around 1°C, after 45 min they were transferred to water of around 15°C, and after another 45 min they were transferred to water of around 0°C. The data are illustrated in the following figure. The gray dots are CTD measurements; the 7 black lines are the measured temperatures from the Acousondes.

It is clear how the Acousonde values decay exponentially toward the true temperature and that it takes about 15 min for the Acousondes to stabilize at the new temperature. We therefore applied the following model. Let *X_t_* denote the measured temperature at time *t* of a given Acousonde, and let *μ_t_* denote the true temperature at time *t* as measured by the CTD. Then, we assume that *X_t_* follows an Ornstein–Uhlenbeck process with a time varying mean of *μ_t_*_ +_ *_α_*, where *α* is an offset modeling a possible bias term, with decay rate *β*, and infinitesimal variance *σ* ^2^. Here, *β*, *α*, and *σ* ^2^ are parameters to be estimated for each Acousonde. Thus, the model is a stochastic differential equation (SDE), where *X_t_* is the solution to the SDE

$$dX_{t}=-\beta \left(X_{t}-\left(\mu_{t}+\alpha \right)\right)dt+\sigma dW_{t}$$

where *dW_t_* are increments of a Brownian motion. We observe *X_t_* and *μ_t_* every 30 s, at time points *t_i_* = *i*∆ with ∆ = 30 s. We will write *X_t_* = *X_i_* and likewise for *μ_t_*. Then, the above model implies that *X_i_*_ + 1_ = *X_i_e^−β^* ^∆^ + (*μ_i_* + *α*)(1 − *e^−β^* ^∆^) + *ξ_i_*_ + 1_ where the *ξ_i_*'s are independent and normally distributed with mean 0 and variance *σ* ^2^(1 – e^−2^ *^β^* ^∆^). The parameters can then be estimated by maximum likelihood, yielding the following estimates for the 7 Acousondes.

**Table:**

Acousonde	*β*	*α*	*σ* ^2^
11	0.00231	0.15910	0.00055
23	0.00244	0.03790	0.00069
26	0.00277	0.01289	0.00093
27	0.00257	0.30709	0.00074
28	0.00245	0.24005	0.00055
31	0.00216	0.02596	0.00053
32	0.00216	0.02596	0.00053

The typical time constants for the delay (the time it takes to decay to around 1/3 of the distance) of the 7 Acousondes in minutes are thus 1/(*β* × 60), with actual values: 7.23, 6.83, 6.02, 6.49, 6.8, 7.73 and 7.73.

Estimates of true temperature given the Acousonde measurements and the estimated parameters

To estimate the true temperature, we use the Kalman filter. The Kalman filter is the standard algorithm to recover an unobserved coordinate from a linear Gaussian process. Thus, in the sequel, we assume μt unknown, and the goal is to recover μt based only on observations *X_i_*, *i* = 1,… *n*. To this end, we assume that the true temperature is a random process with Gaussian increments:

$$\mu_{i+1}=\mu_{i}+\eta_{i-1}$$

where the *η_i_*s are independent and normally distributed with mean 0 and variance ${\tilde{\sigma }}^{2}$ . The variance parameter ${\tilde{\sigma }}^{2}$ is estimated by:

$${\tilde{\sigma }}^{2}=\frac{1}{n-3}\sum_{i=2,i\neq n_{1},n_{2}}^{n}{\left(\mu_{i}-\mu_{i-1}\right)}^{2}$$

where *n*_1_ and *n*_2_ are the times where the Acousondes switched water temperature. We obtain $\tilde{\sigma }$ = 0.00010296. The implementation of the Kalman filter then yields the estimates of the temperature as given in the following figure, where the green lines are the estimated temperatures. Notice that the gray points are not used in the estimation.

The Kalman filter seems to recover well the unknown temperature. In the following, the Kalman filter is applied to the records of four narwhals, with the parameters provided from the above analysis. The only parameter that has to be adapted to the setting of the tagged data is the variance of the true temperature, ${\tilde{\sigma }}^{2}$ . Data from CTD tags attached to other animals in the same areas of East Greenland provide information about how temperature changes during normal diving patterns of narwhals.

The variability in the temperature measurements is very high at the surface (sometimes the temperature changes more than 2°C within one second, which is probably wrong), so only temperatures measured at depths of less than −30 m were used. Notice, however, that these two whales only dived to −200 m and −250 m, respectively, so we have no information about deeper dives. The estimate is $\tilde{\sigma }$ = 0.002.

In the first plot, histograms of measured as well as estimated temperatures are presented. Note how the measured temperatures have a unimodal distribution, whereas the estimated temperatures follow a bimodal distribution. This is in agreement with the whales spending time in warmer waters either at the surface or at the bottom of the dives, and passing through colder waters when diving deep.

The next plots show example traces of 1 hr of measured and estimated temperatures together with depth. The black lines are the depths (scale on left axis), the green lines are the measured, and the blue lines are the estimated temperatures (scale on right axes). The dashed gray lines are at temperature 0°C, to show how temperature estimates are positive at the bottom of the dives, and only negative for short times when passing through the water column.

The last plots show the distribution of temperatures at different depths. Gray points are the measured temperatures, black points are the estimated temperatures, and the green curve is the average temperature through the water column in these waters.

### Reference

Fernando, Tusell (2011). Kalman Filtering in R. Journal of Statistical Software, 39(2).
